# Study on mechanism of improving efficiency of permanent-magnet small ball-end magnetorheological polishing by increasing magnetorheological fluid temperature

**DOI:** 10.1038/s41598-022-11937-8

**Published:** 2022-05-11

**Authors:** Jinchuan Tian, Mingjun Chen, Henan Liu, Biao Qin, Jian Cheng, Yazhou Sun

**Affiliations:** Center for Precision Engineering, Harbin Institute of Technology, P.O. Box 413, Harbin, 150001 Heilongjiang People’s Republic of China

**Keywords:** Engineering, Aerospace engineering, Mechanical engineering

## Abstract

Permanent-magnet small ball-end magnetorheological polishing method can be used to polish the small part with complex structure. However, the material removal rate of this method is low, which is difficult to improve the output and reduce the cost. In this research, the effect of magnetorheological fluid temperature on the material removal rate is theoretically analyzed by measuring the effect of temperature on the flow properties of magnetorheological fluid, establishing the hydrodynamic model of polishing zone and solving the material removal parameters. It is found that with the increase of the magnetorheological fluid temperature, the polishing relative velocity increases accordingly, which can promote the improvement of material removal rate. But the shear stress decreases accordingly, which inhibits the improvement of material removal rate. The verification experiment results show that the promoting effect can exceeds the inhibitory effect, so that the material removal rate increases with the increase of magnetorheological fluid temperature. When the magnetorheological fluid temperature increases to 60 °C, the material removal rate is improved by 108.4% and the polished surface roughness Sa can reach 14.9 nm. Therefore, increasing the magnetorheological fluid temperature can significantly improve the efficiency of permanent-magnet small ball-end magnetorheological polishing and obtain high quality polished surface.

## Introduction

The small part with complex structure plays an important role in all kinds of precision equipment. These parts are mostly made of hard and brittle materials that are difficult to process. The polished surface quality and profile accuracy of these parts are required highly. Magnetorheological (MR) polishing method has the advantages of high machining accuracy, no tool wear and no subsurface damage, and the roughness of polished surface can reach nanoscale^1,2^ or even angstrom dimension^3^. Thus, it is suitable for polishing these parts. However, the size of the polishing wheel of the commonly used wheel-type MR polishing equipment is too large, which cannot polish the complex structure surface of the small part. Therefore, the small MR polishing tool is needed to be designed and used. Chen et al.^4^ designed a permanent-magnet small ball-end polishing head (diameter 4 mm) and successfully polished a Ψ-shaped small-bore complex component. The minimal transition fillet curvature radius of the curved surfaces of the component was less than 3 mm. The surface accuracy PV of polished surface reached 0.332 μm, and the surface roughness Ra reached 10.7 nm. However, limited by the size of the polishing head, the volume of the permanent magnetic material of the polishing head is small, leading to the relatively low magnetic induction intensity (no more than 0.44 T). Besides, the linear velocity of the polishing head at high rotation speed is low, which limits the improvement of polishing relative speed. These factors result in low material removal rate during polishing. The processing cost is high and the output is low. Therefore, it is urgent to improve the efficiency of the permanent-magnet small ball-end MR polishing.

Ultrasonic vibration^5–7^, Non-resonant vibration^8^ and chemical action^9–12^ are introduced into the MR polishing process, which can significantly improve the material removal rate and polished surface quality. However, there are few studies on improving the material removal rate by changing the MR fluid temperature. MR fluid is a typical non-Newtonian fluid. Its flow properties are closely related to temperature^13,14^. Hemmatian et al.^15^ studied the temperature dependency of MR fluids’ properties. It was found that the effect of temperature on the viscosity and shear stress of MR fluid diminished with increasing magnetic field. Wang et al.^16^ and Sherman et al.^17^ studied the temperature-dependent material properties of the components of MR fluids. It was found that the viscosity of MR fluid depended on the viscosity of carrier fluid. The viscosity of the carrier fluid decreased as the temperature increased, and the carrier fluid with a higher viscosity was more sensitive to the temperature variation. Chen et al.^18^ analyzed the influence of temperature on the rheological properties of MR fluid. It was found that within 100 °C, the viscosity of MR fluid decreased with the increase of temperature. The shear stress of MR fluid was affected by the change of viscosity and would decrease with the increase of temperature. Wang et al.^19^ used a parallel disk shear stress testing device to measure the temperature-dependent mechanical properties of MR fluids. It was found that with the increase of MR fluid temperature, the reduction in viscous stress was much more evident than that in yield stress. The viscous stress component dominated the change of total stress in a particular temperature range. Bahiuddin et al.^20^ used the extreme learning machine (ELM) method to develop a new constitutive model of MR fluids with temperature-dependent prediction parameter. It accurately predicted the shear and yield stresses of MR fluids under specific temperature, shear rate and magnetic field. In conclusion, changing the MR fluid temperature can influence the flow properties and mechanical properties, so as to affect the polishing removal efficiency.

Aiming at the permanent-magnet small ball-end polishing method, the influence law of temperature on MR fluid flow properties and material removal parameters are studied, and the verification experiment is carried out in this research. The experiment results show that increasing the MR fluid temperature can greatly improve the polishing removal efficiency.

## Material removal mechanism

**Figure 1:**
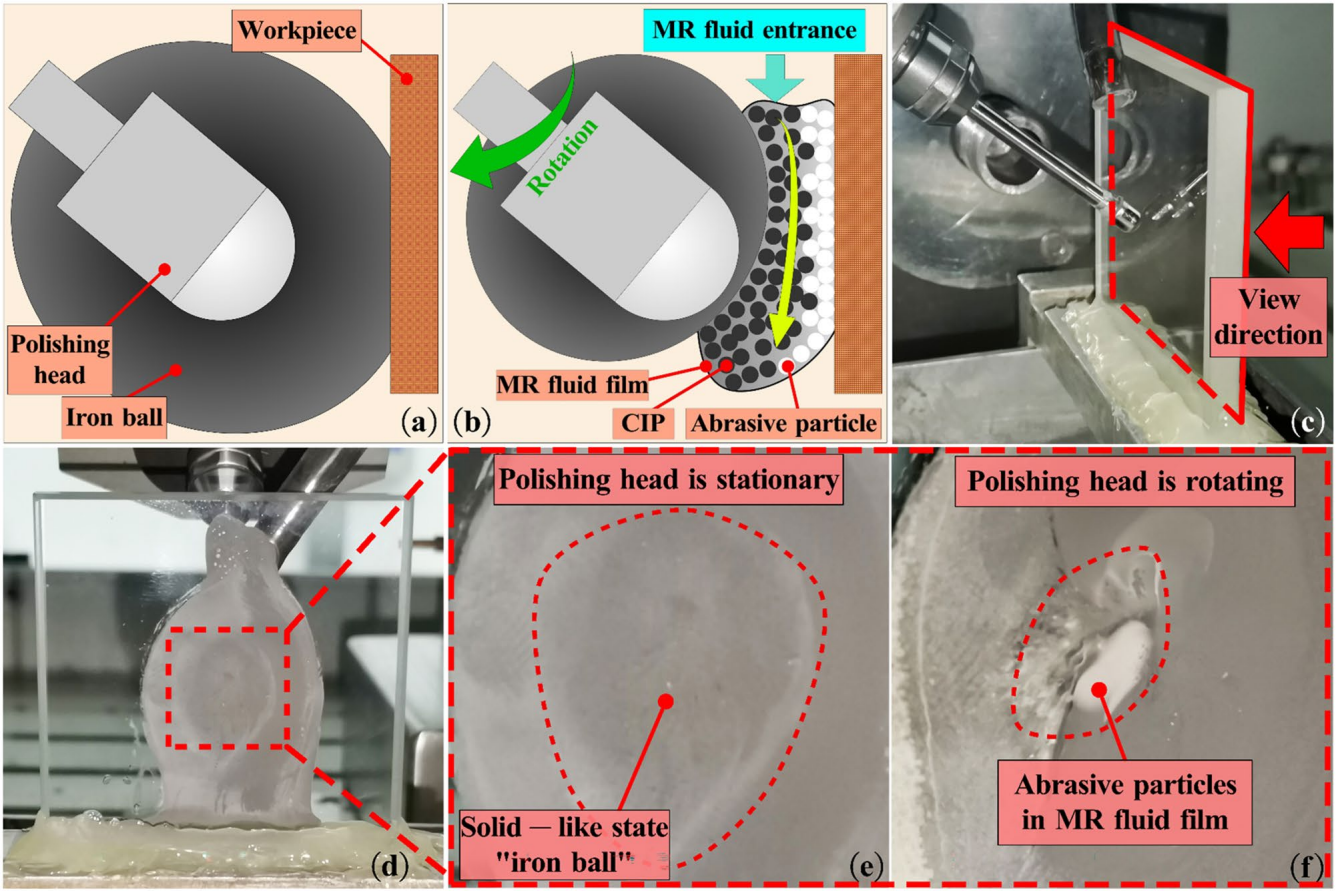
Schematic diagram of material removal mechanism. (**a**) Polishing zone when the polishing head is stationary; (**b**) Polishing zone when the polishing head is rotating; (**c**) View direction; (**d**) Back of workpiece surface; (**e**) Iron ball with solid-like state; (**f**) MR fluid film.

In this research, the solid particles in water-based MR fluid are carbonyl iron powder (CIP) and cerium oxide abrasive particle. When the polishing head is stationary, the MR fluid flowing into the polishing zone is affected by the applied magnetic field of the polishing head. The MR fluid is adsorbed on the surface of the polishing head to form an "iron ball" with solid-like state, as shown in Fig. 1a. During the high-speed rotation of the polishing head, the iron ball rotates synchronously with the polishing head to generate shear stress between the iron ball and the workpiece surface. When the shear stress is greater than the yield stress of MR fluid, the MR fluid in contact with the workpiece surface changes from solid-like state to flowing state, forming a flowing "MR fluid film", as shown in Fig. 1b. In the polishing process, fresh MR fluid is continuously supplied to the polishing zone, which can form the continuous flow of MR fluid film between the iron ball and the workpiece surface. Under the action of magnetic field, the CIPs in the MR fluid film are distributed close to the polishing head surface, so as to occupy the space of non-ferromagnetic abrasive particles and push the abrasive particles towards the workpiece surface far away from the polishing head. This not only makes the abrasive particles subject to the normal force perpendicular to the workpiece surface, but also greatly improves the abrasive particle concentration on workpiece surface. With the flow of MR fluid film and the action of normal force, these abrasive particles continuously shear the workpiece surface at a certain speed, so as to realize material removal. The iron ball and MR fluid film on the workpiece surface were observed through the experiment. The observation direction is shown in Fig. 1c,d, the iron ball on the workpiece surface is shown in Fig. 1e, and the flowing MR fluid film is shown in Fig. 1f.

In a certain temperature range, increasing the MR fluid temperature can reduce the initial viscosity of MR fluid, which can reduce the flow resistance of MR fluid film. It is speculated that this can increase the flow velocity of MR fluid film, which can increase the relative velocity when abrasive particles shear the workpiece surface. Moreover, the number of abrasive particles flowing through the workpiece surface per unit time also increases, which can improve the shear frequency between abrasive particles and the workpiece surface, so as to improve the polishing removal efficiency.

## Effect of temperature on flow properties of MR fluid

MR fluid is a non-Newtonian fluid, and the flow properties of MR fluid mainly depend on the initial viscosity and the yield stress. When subjected to an applied magnetic field, the magnetization intensity of MR fluid also affects the flow properties. Therefore, it is necessary to study the influence of temperature on initial viscosity, yield stress and magnetization intensity of MR fluid. In the polishing process, the heated MR fluid firstly transfers heat to the iron ball, and then the iron ball transfers heat to the polishing head. This increases the temperature of the polishing head. The permanent magnetic material of the polishing head is sintered NdFeB. When the temperature increases by 1 °C, the magnetic induction intensity of NdFeB reduces by about 0.11–0.12%^21^. When the temperature exceeds 80 °C, the magnetic field of NdFeB declines irreversibly or even disappears. Based on the laboratory room temperature and the above factors, 20–60 °C is selected as the temperature range of MR fluid. When the temperature rises from 20 to 60 °C, the maximum magnetic induction intensity of the polishing head decreases from 0.439 to 0.418 T. Thus, it is also necessary to consider the influence of the changed magnetic induction intensity of the polishing head on the flow properties of the MR fluid.

### Effect of temperature on initial viscosity

**Figure 2:**
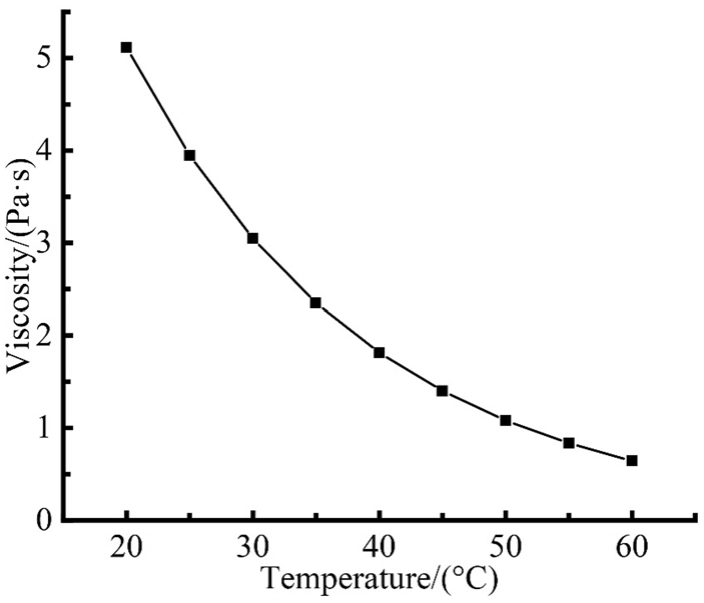
Viscosity-temperature variation curve of MR fluid.

In the absence of magnetic field, the relationship between the initial viscosity and temperature can be expressed by Eq. (1)^22^. The viscosity variation curve in the range of 20–60 °C is shown in Fig. 2.

1 $$ \eta_{0} = 4.573e^{ - 0.05182t} (1 + 2.5\phi + 6.25\phi^{2} ) $$

where *η*_0_ is the initial viscosity of MR fluid (Pa s), *t* is the MR fluid temperature (°C), *ϕ* is the volume percentage of solid particles in MR fluid.

When the MR fluid temperature increases from 20 to 30 °C, 40 °C, 50 °C and 60 °C respectively, the initial viscosity of MR fluid decreases by 40.4%, 64.5%, 78.9% and 87.4% respectively. The results indicate that the initial viscosity of MR fluid is very sensitive to temperature variation. With the increase of temperature, the initial viscosity of MR fluid decreases remarkably. The flow resistance of MR fluid film decreases, which is conducive to increasing the polishing relative velocity between abrasive particles and workpiece surface, so as to improving the material removal rate.

### Effect of temperature on yield stress

After the yielding flow is generated, the flow state of MR fluid can be expressed by Bingham model, as shown in Eq. (2).

2 $$ \tau  = \tau_{0} { + }\eta_{0} \dot{\gamma } $$

where *τ* is shear stress (Pa), *τ*_0_ is yield stress (Pa), *η*_0_ is initial viscosity (Pa s), $\dot{\gamma }$ is shear rate (s^−1^).

**Figure 3:**
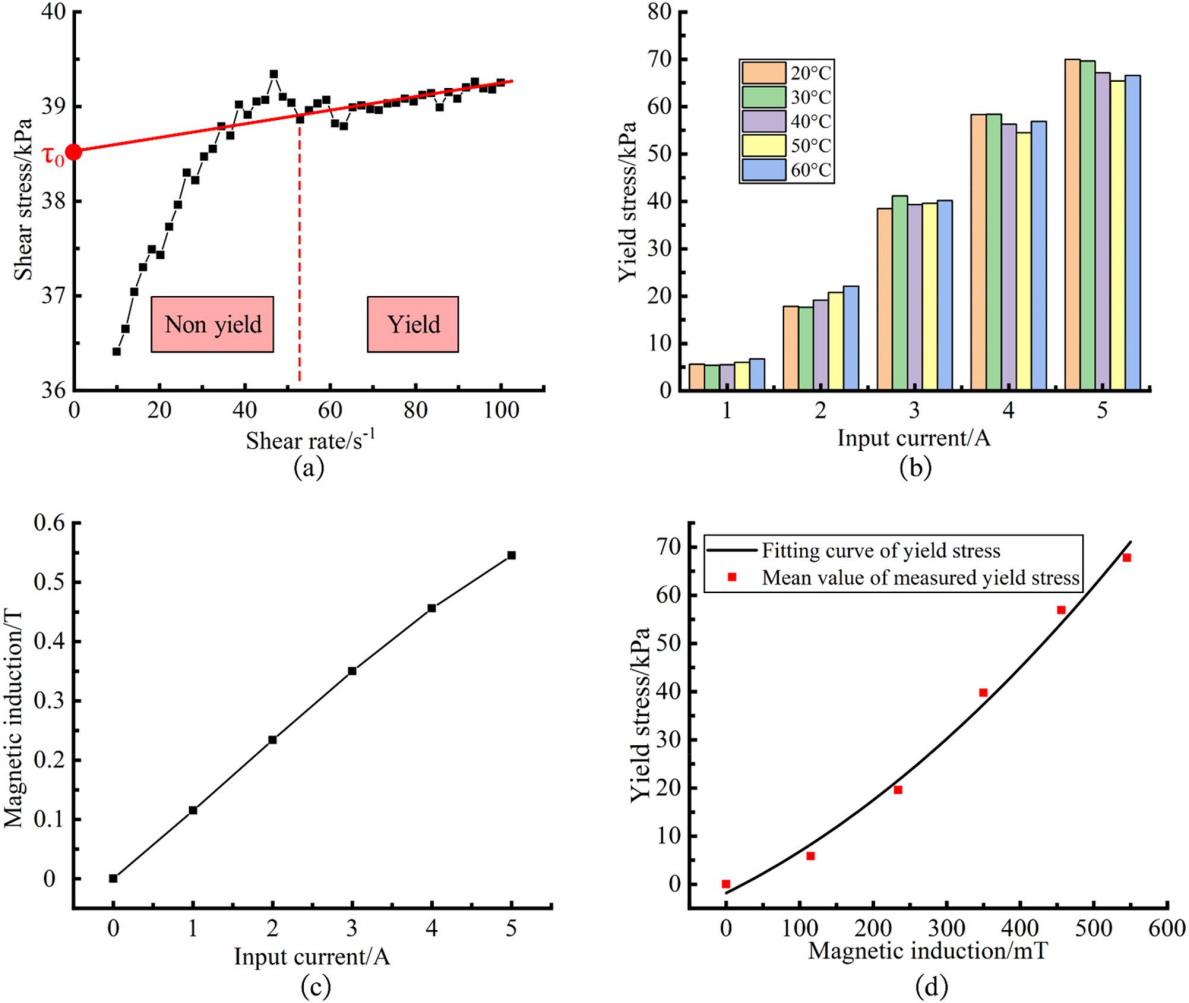
Schematics of measurement and calculation of yield stress. (**a**) The typical shear rate vs. shear stress curve; (**b**) The variation trend of yield stress; (**c**) The relationship between input current and magnetic induction intensity; (**d**) The curve of the yield stress fitting model.

The shear rate vs. shear stress curve of MR fluid was measured by Anton Paar MCR301 rheometer, and the magnetic induction intensity of the applied magnetic field was depended on the input current of the magneto-rheological device of the rheometer. The curve was measured repeatedly by changing the temperature and input current. A typical shear rate vs. shear stress curve is shown in Fig. 3a (MR fluid temperature 20 °C, input current 3A). With the increase of shear rate, the MR fluid is transformed from non-yield state to yield flow state. The Bingham model is used to linearly fit the shear rate-shear stress data after yield. The longitudinal intercept of the fitting line is the yield stress of MR fluid, as shown in Fig. 3a. The yield stress at different temperatures and different input current is shown in Fig. 3b, and the relationship between magnetic induction intensity of the applied magnetic field and input current is shown in Fig. 3c. The mean value of measured yield stress under each input current is shown in Fig. 3d.

It can be seen from Fig. 3b that the yield stress varies less with temperature. Under the same input current, the variation range of yield stress with temperature is 1.4–4.5 kPa. However, under the same temperature, the variation range of yield stress with input current is 59.4–64.3 kPa. It suggests that the yield stress is less affected by temperature variation and more sensitive to the variation of magnetic induction intensity of applied magnetic field, which is consistent with the research results in reference^15^ and reference^19^. In order to simply the calculation, it is considered that the yield stress is only relate to the magnetic induction intensity of the polishing head. The mathematical model of yield stress is obtained by fitting, as shown in Eq. (3). The curve of the mathematical model is shown in Fig. 3d.

3 $$ \tau_{0} (B_{0} ) = 1.0287 * 10^{ - 4} B_{0}^{2} + 0.0759B_{0} - 1.8081 $$

where *τ*_0_ is the yield stress of MR fluid (kPa), *B*_0_ is the magnetic induction intensity of the polishing head (mT).

In the polishing process, the magnetic induction intensity of the polishing head decreases with the increase of the MR fluid temperature, resulting in the reduce of the yield stress. According to Eq. (2), under the same initial viscosity and shear rate, the shear stress decreases with the decrease of yield stress, which is not beneficial to improve the material removal rate.

### Effect of temperature on magnetization intensity of CIP

**Figure 4:**
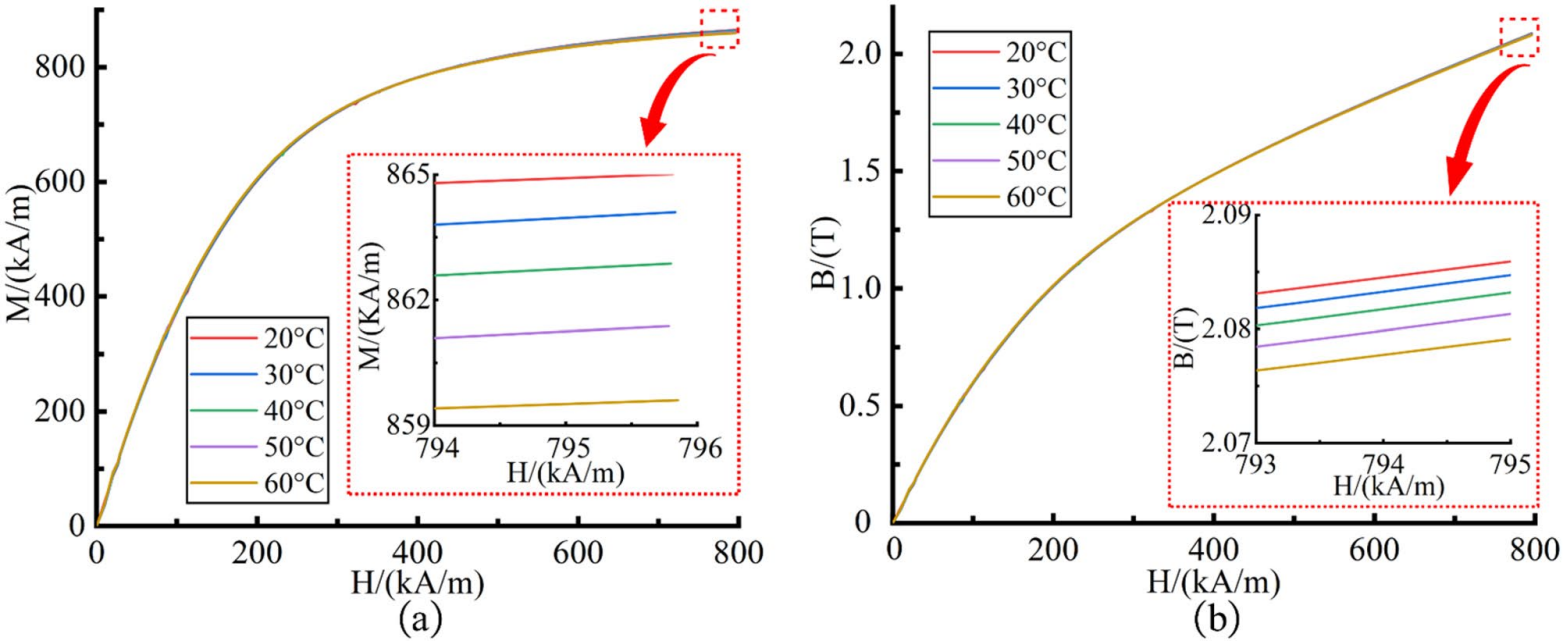
The magnetization properties curves of CIP. (**a**) The magnetization intensity curves at different temperatures; (**b**) The *B-H* curves at different temperatures.

The magnetization intensity of CIP is related to temperature. Increasing the temperature can change the magnetization intensity of CIP, thus affecting the flow properties of MR fluid. Therefore, it is necessary to study the effect of temperature on CIP magnetization intensity. The magnetization intensity curve of CIP in the range of 20–60 °C is measured by the Physical Property Measurement System (PPMS), as shown in Fig. 4a.

It can be seen from Fig. 4a that the magnetization intensity of CIP increases with the increase of applied magnetic field intensity. In the applied magnetic field intensity range of 0–300 kA/m, the magnetization intensity of CIP increases sharply. With the further increase of the applied magnetic field intensity, the rising trend of magnetization intensity gradually slows down until it reaches the maximum value within the test range. In the range of 20–60 °C, the magnetization intensity curves are highly coincident. Under the same applied magnetic field intensity, the magnetization intensity decreases with the increase of temperature, but the decrease of magnetization intensity can be ignored. For example, when the applied magnetic field intensity is 795 kA/m, the increase of temperature from 20 to 60 °C reduces the magnetization intensity by 5.4 kA/m. The reduction amplitude only accounts for 0.63% of the minimum magnetization intensity. This suggests that the magnetization intensity of CIP has good stability in this temperature range. In the polishing process, it is unlikely to affect the flow properties by changing the magnetization intensity.

Moreover, the total magnetic field in the polishing zone consists of an applied magnetic field of polishing head and an induced magnetic field generated by CIP. It can be seen from Eq. (4) that the change of CIP magnetization intensity with temperature can affect the total magnetic induction intensity in the polishing zone. This can change the yield stress of MR fluid, thus affecting the material removal rate.

4 $$ B = \mu_{0} (H + M) $$

where *B* is the total magnetic induction intensity (T), *H* is the applied magnetic field strength of polishing head (A/m), *M* is the magnetization intensity of CIP (A/m), *μ*_0_ is the permeability of vacuum (N/A^2^).

Thus, it is also necessary to study the effect of CIP magnetization intensity on the total magnetic induction intensity at different temperatures. The *B-H* curves of CIP at different temperatures are obtained by solving the Eq. (4), as shown in Fig. 4b. Under the same applied magnetic field intensity, the change of total magnetic induction intensity with temperature is negligible. It can be considered that the applied magnetic field intensity determines the total magnetic induction intensity. This shows that in the range of 20–60 °C, the change of total magnetic induction intensity caused by the change of magnetization intensity is little, which is unlikely to have a significant impact on the yield stress. This is consistent with the phenomenon that the yield stress varies less with temperature found in Fig. 3b. This result suggests that the change of CIP magnetization intensity at different temperatures is not the main reason affecting the material removal rate.

In conclusion, increasing the MR fluid temperature can reduce the initial viscosity of MR fluid. The fluidity of MR fluid film can be improved, which is conductive to improving the material removal rate. The change of CIP magnetization intensity at different temperatures is not the main reason affecting the material removal rate. The magnetic field intensity of the polishing head decreases with the increase of temperature. The total magnetic induction intensity in the polishing zone also decreases, resulting in the reduction of the yield stress. This may reduce the shear stress of MR fluid, which is not conducive to improving the material removal rate. Therefore, the change rule of material removal rate with temperature is still unclear, which needs further quantitative analysis.

## Calculation and analysis of material removal parameters of polishing zone

Dorier^23^ proposed a modified Bingham model, as shown in Eq. (5). In this model, the non-yield fluid is regarded as the slow flowing fluid with extremely high viscosity, which make the boundary of the yield zone and non-yield zone transit continuously and smoothly. Therefore, the model can be directly used to calculate the flow parameters of MR fluid in the polishing zone without judging the yield state of the MR fluid in advance.

5 $$ \tau  = \eta_{0} \dot{\gamma }{ + }\frac{{2\tau_{0} }}{\pi }\tan^{ - 1} \left( {\frac{{\dot{\gamma }}}{{\dot{\gamma }_{0} }}} \right) $$

where *τ* is the shear stress (Pa), *η*_0_ is the initial viscosity (Pa s), $\dot{\gamma }_{0}$ is the initial shear rate (s^−1^), $\dot{\gamma }$ is the shear rate (s^−1^), *τ*_0_ is the yield stress (Pa).

Equation (5) can be converted to the definition of apparent viscosity of MR fluid, as shown in Eq. (6).

6 $$ \eta (\dot{\gamma }) = \eta_{0} { + }\frac{{2\tau_{0} }}{{\pi \dot{\gamma }}}\tan^{ - 1} \left( {\frac{{\dot{\gamma }}}{{\dot{\gamma }_{0} }}} \right) $$

Combine Eqs. (1), (3) and (6) to obtain Eq. (7).

7 $$ \begin{aligned} \eta (\dot{\gamma }) &= 4.573e^{ - 0.05182t} (1 + 2.5\phi + 6.25\phi^{2} ) \hfill \\ &\quad+ \frac{{2(1.0287 * 10^{ - 4} B_{0}^{2} + 0.0759B_{0} - 1.8081)}}{{\pi \dot{\gamma }}}\tan^{ - 1} \left( {\frac{{\dot{\gamma }}}{{\dot{\gamma }_{0} }}} \right) \hfill \\ \end{aligned} $$

**Figure 5:**
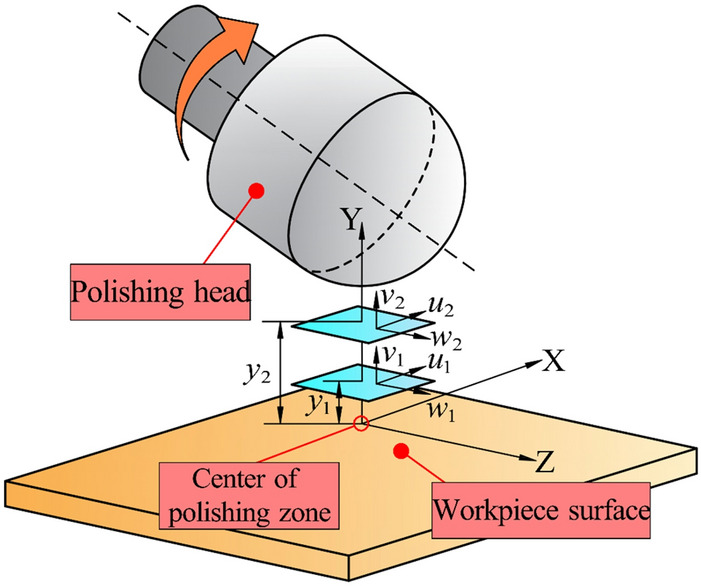
Schematic diagram of MR fluid hydrodynamic analysis model and coordinate system.

The MR fluid in the polishing zone is divided into many small three-dimensional meshes. The flow parameters of each node of the meshes are soled by the finite difference method, so as to obtain the flow state of the whole polishing zone. In order to simplify the calculation, a smooth and stationary plane is regarded as the workpiece surface. The hydrodynamic analysis model and the coordinate system of MR fluid are shown in Fig. 5. The Y axis passes through the spherical center of the hemisphere of the polishing head and is perpendicular to the workpiece surface. The rotation axis of the polishing head is perpendicular to the X axis in space and intersects with the Z axis, and the included angle is 40°. The center of the polishing zone is the zero point of X, Y and Z axes on the workpiece surface. In the flowing process, the upper wall of MR fluid is the polishing head surface, and the lower wall is the workpiece surface. The flow velocities of MR fluid along X, Y and Z directions are *u*, *v* and *w* respectively.

The following assumptions are made for the MR fluid.

1. The MR fluid is an incompressible fluid with constant density;
2. The flow of MR fluid between the upper and lower walls is laminar flow and meets the no-slip condition, hence the flow velocity of the MR fluid at the boundary is equal to the velocity of the solid surface;
3. The gravity and inertia force of the MR fluid are ignored;
4. The partial derivatives of flow velocity and stress in X and Z directions are ignored;
5. The hydrodynamic pressure across the film thickness remains constant.

The boundary conditions of MR fluid are: *y*_1_ = 0, *u*_1_ = *v*_1_ = *w*_1_ = 0; *y*_2_ = *d*, *u*_2_ = *U*_2_, *v*_2_ = *V*_2_, *w*_2_ = *W*_2_. The boundary conditions are substituted into the velocity expression, fluid dynamic pressure expression and apparent viscosity expression of MR fluid to obtain Eqs. (8–10)^24^.

8 $$ u = \frac{\partial P}{{\partial x}}\left[ {\frac{{y^{2} - yd}}{{2\eta (\dot{\gamma })}}} \right] + \frac{{yU_{2} }}{d} $$

9 $$ w = \frac{\partial P}{{\partial z}}\left[ {\frac{{y^{2} - yd}}{{2\eta (\dot{\gamma })}}} \right] + \frac{{yW_{2} }}{d} $$

10 $$ \frac{\partial }{\partial x}\frac{{U_{2} d}}{2} + \frac{\partial }{\partial z}\frac{{W_{2} d}}{2} - \frac{\partial }{\partial x}\left( {\frac{\partial P}{{\partial x}}\frac{{d^{3} }}{{12\eta (\dot{\gamma })}}} \right) - \frac{\partial }{\partial z}\left( {\frac{\partial P}{{\partial z}}\frac{{d^{3} }}{{12\eta (\dot{\gamma })}}} \right) = \frac{\partial d}{{\partial x}}U_{2} + \frac{\partial d}{{\partial z}}W_{2} - V_{2} $$

The flow parameters of MR fluid are calculated by iteratively solving Eqs. (7–10) by finite difference method.

### Analysis of material removal parameters

**Figure 6:**
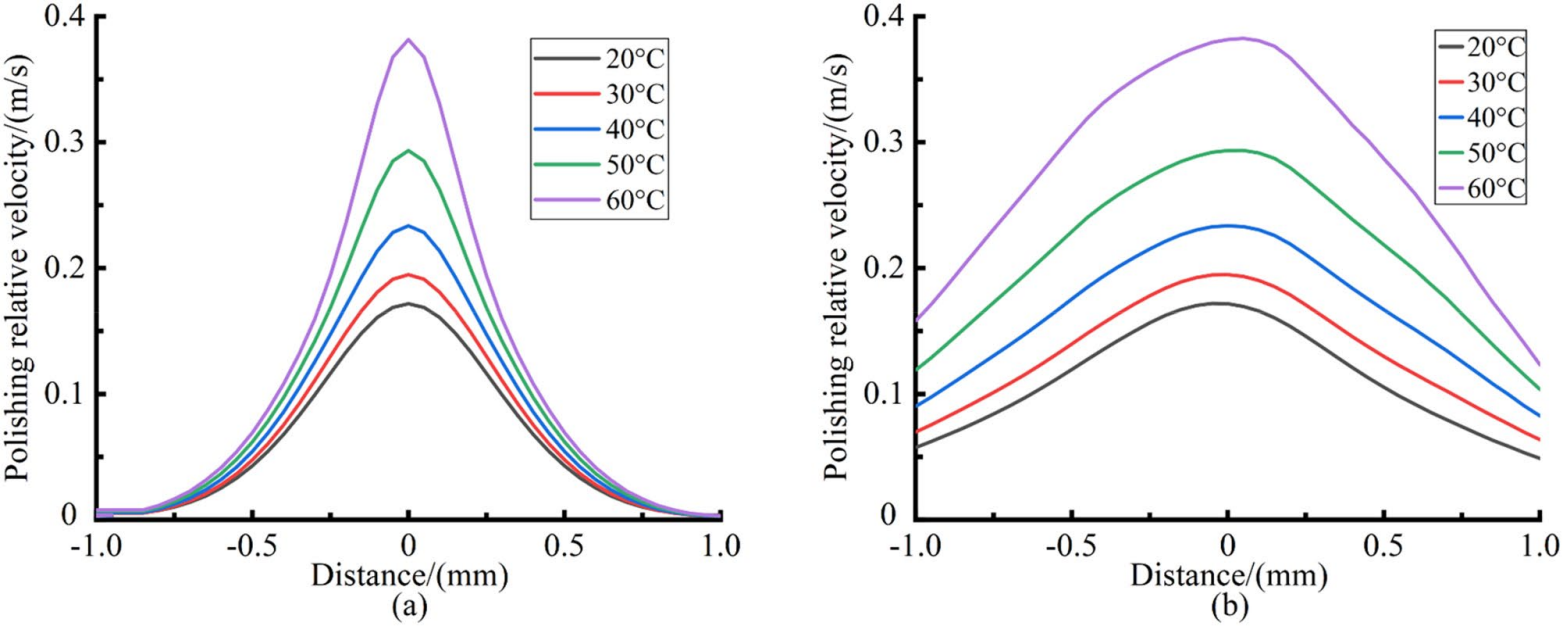
Polishing relative velocity distribution curves. (**a**) Along the X axis; (**b**) Along the Z axis.

**Figure 7:**
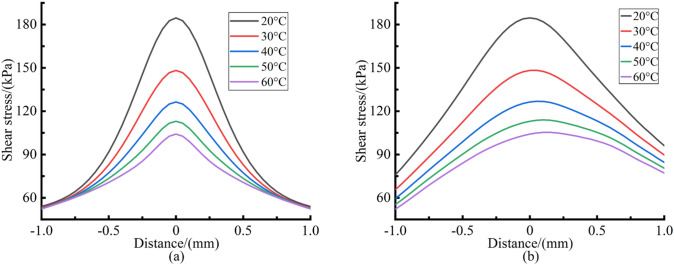
Shear stress distribution curves. (**a**) Along the X axis; (**b**) Along the Z axis.

The average particle size of CIP is 7–8 μm, and the average particle size of abrasive particles is 8–10 μm. Therefore, it can be assumed that the MR fluid flow velocity and shear stress at 8 μm above the workpiece surface are the polishing relative velocity and the shear stress that determine the material removal during polishing process. A set of processing parameters commonly used in polishing process are as follows: polishing gap is 0.1 mm and polishing head rotation speed is 7000 rpm. Taking this processing parameters as an example, the polishing relative velocity and the shear stress along the X axis and Z axis during the material removal process were calculated by solving Eqs. (7–10), as shown in Figs. 6 and 7 respectively.

It can be seen from Figs. 6 and 7 that with the increase of MR fluid temperature, the polishing relative velocity continues to increase and the shear stress continues to decrease. As the MR fluid temperature increases from 20 to 60 °C, the maximum polishing relative velocity is increased by 13.3%, 35.9%, 70.7% and 122.5% respectively, and the maximum shear stress is decreased by 19.7%, 31.3%, 38.2% and 42.9% respectively. This is because the increase of temperature reduces the viscosity of the carrier fluid. The viscous resistance decreases when the MR fluid flows, thus the fluidity of MR fluid becomes better. The polishing relative velocity between abrasive particles and workpiece surface is improved, which is conducive to improving the material removal rate. The yield stress of MR fluid reduces with the decrease of the total magnetic induction intensity in the polishing zone, leading to the decrease of shear stress. Moreover, the lower the viscosity of the carrier fluid, the more significant the lubrication effect on the micron particles in the carrier fluid. This may reduce the interaction force between particles in MR fluid, thus reducing the shear stress.

### Distribution zone of material removal rate

In the process of MR polishing, the shear stress plays a major role in material removal^25,26^. The Preston equation based on shear stress can be used to establish the material removal rate model of MR polishing, as shown in Eq. (11).

11 $$ MRR = K\tau V $$

where *MRR* is the material removal rate (m^3^/s), *K* is coefficient of correction (m^2^/kPa), *τ* is the shear stress (kPa), *V* is the polishing relative velocity (m/s).

**Figure 8:**
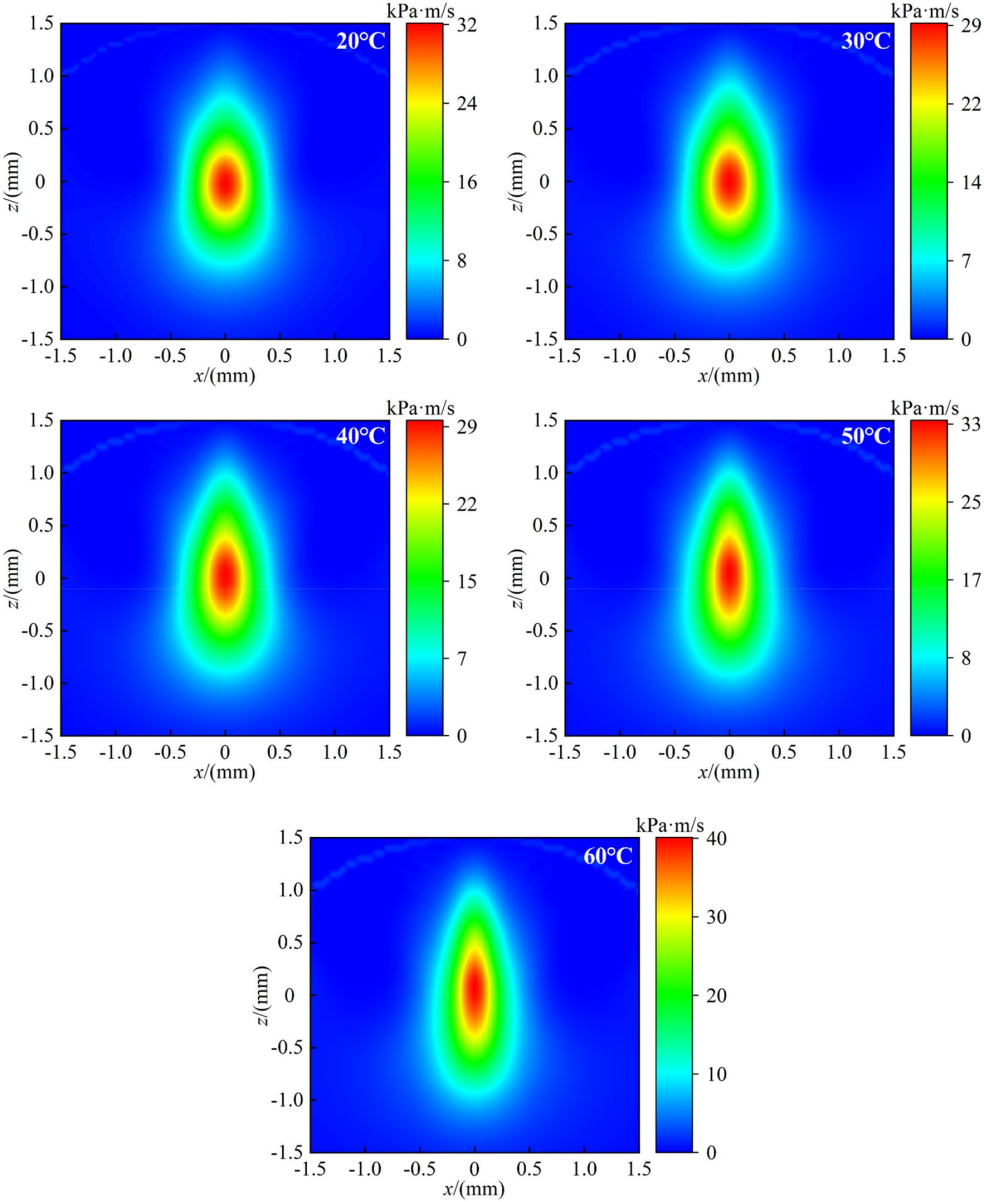
Product distribution zone of relative velocity and shear stress.

Ignoring the coefficient of correction, the product of shear stress and polishing relative velocity can reflect the distribution zone of the material removal rate on the polished surface. Taking the MR fluid temperature at 20–60 °C as examples, the product of shear stress and polishing relative velocity on the polished surface were calculated respectively, as shown in Fig. 8.

It can be seen from Fig. 8 that after raising the MR fluid temperature, the position of maximum material removal rate is still at the center of the distribution zone. The closer to the edge of the distribution zone, the lower the material removal rate. It is conducive to maintaining the convergence of surface shape accuracy in material removal process. According to the Eq. (11), the increase of polishing relative velocity has a promoting effect on improving the material removal rate, but the decrease of shear stress has an inhibitory effect. Therefore, the improvement of material removal rate needs to be verified by experiments.

## Verification experiment

**Figure 9:**
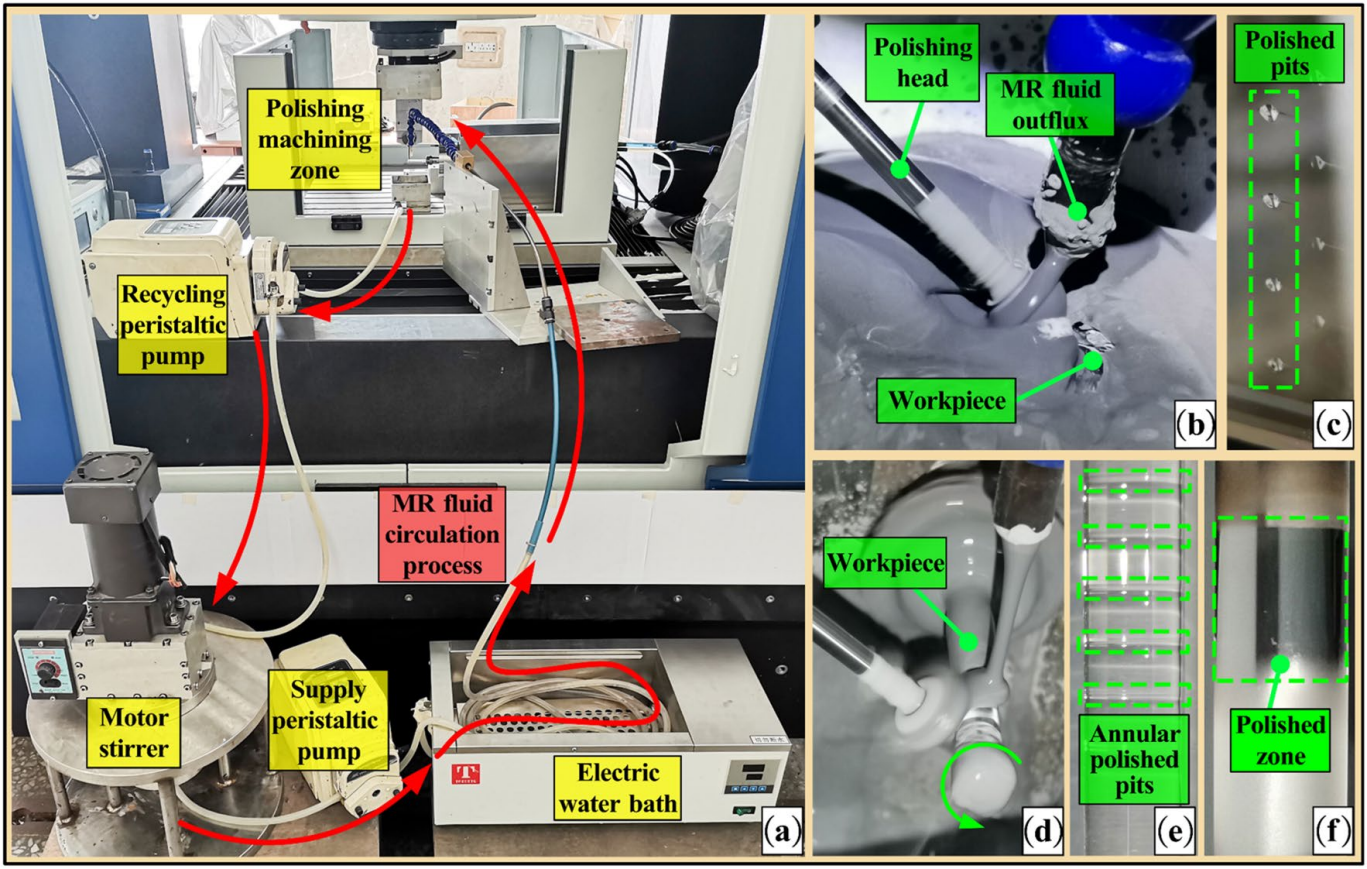
Schematic diagram of polishing experiment. (**a**) MR fluid circulation process and experimental equipment; (**b**) Plane glass polishing experiment; (**c**) Teardrop-shaped polished pits; (**d**) Glass rod polishing experiment; (**e**) Annular polished pits; (**f**) Polished smooth surface.

The polishing experimental equipment and MR fluid circulation process are shown in Fig. 9a. The hose used to convey MR fluid is coiled into loops and put into a water bath kettle. A probe thermometer is used to measure the MR fluid temperature at the outflux. The MR fluid temperature is adjusted to the target temperature of experiment by adjusting the set temperature of water bath kettle. In order to verify the effect of MR fluid temperature on the position of maximum material removal rate, the fixed-point polishing experiment was carried out on a plane glass workpiece, as shown in Fig. 9b. The teardrop-shaped pits obtained by polishing are shown in Fig. 9c. In order to measure the material removal volume of the polished pit conveniently and accurately, the fixed-point polishing experiment was carried out on a K9 glass rod with length of 50 mm and diameter of 6 mm, as shown in Fig. 9d. The annular polished pits on the surface of the glass rod are shown in Fig. 9e.

**Figure 10:**
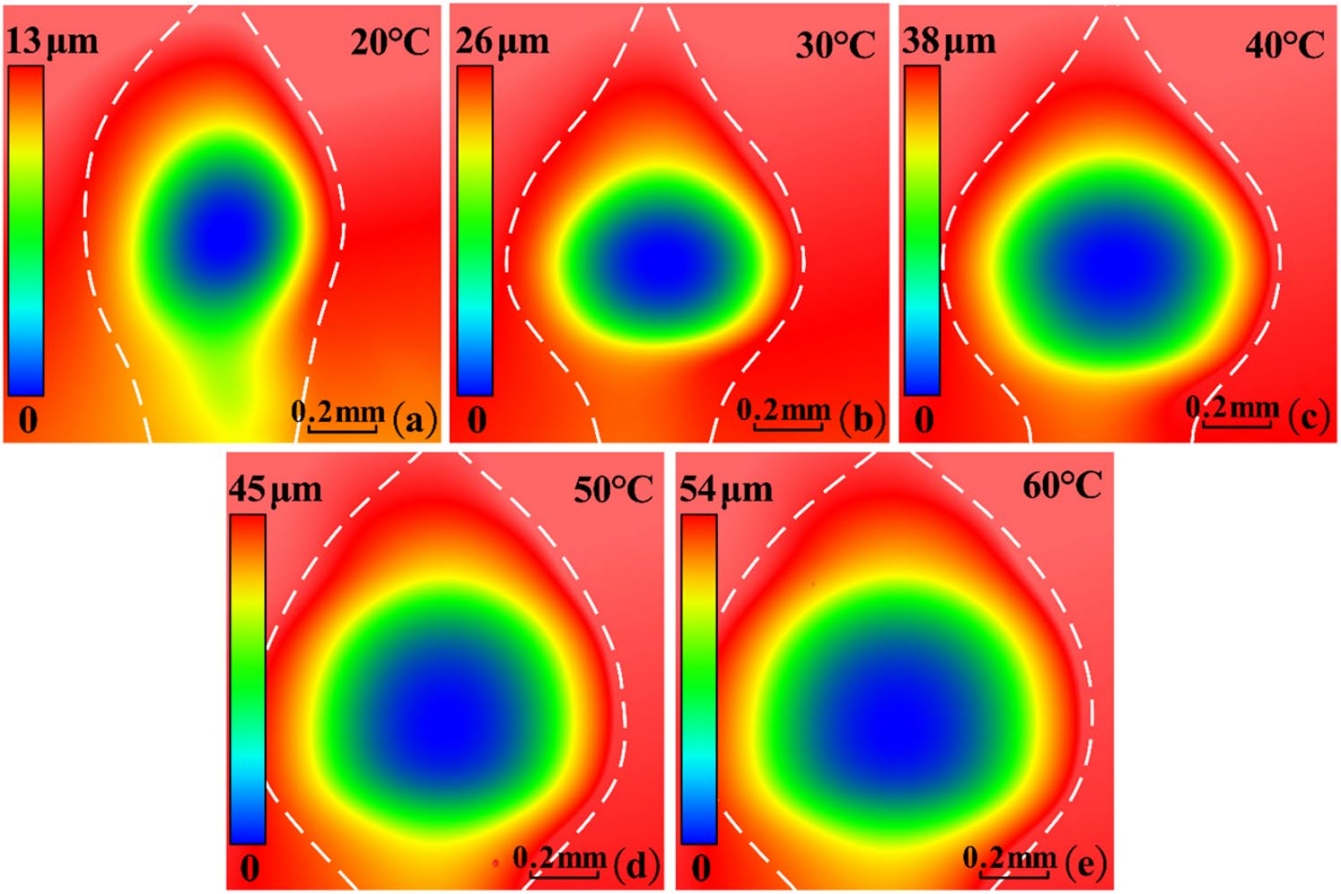
The shape of teardrop-shaped polished pits. (**a**) 20 °C; (**b**) 30 °C; (**c**) 40 °C; (**d**) 50 °C; (**e**) 60 °C.

The processing parameters are as follows: MR fluid temperature is 20–60 °C, polishing head rotation speed is 7000 rpm, polishing gap is 0.1 mm. When polishing the plane glass workpiece, the polishing time of each pit is 5 min. When polishing the glass rod workpiece, the workpiece rotation speed is 120 rpm and the polishing time of each pit is 20 min. In the polishing process, the water of the MR fluid is replenished every 10 min to keep the mixture ratio of MR fluid stable. A Zygo NewView 8200 white-light interferometer was used to measure the three-dimensional shape and the section contour of the polished pit. The shape of teardrop-shaped polished pits at different MR fluid temperatures is shown in Fig. 10.

**Figure 11:**
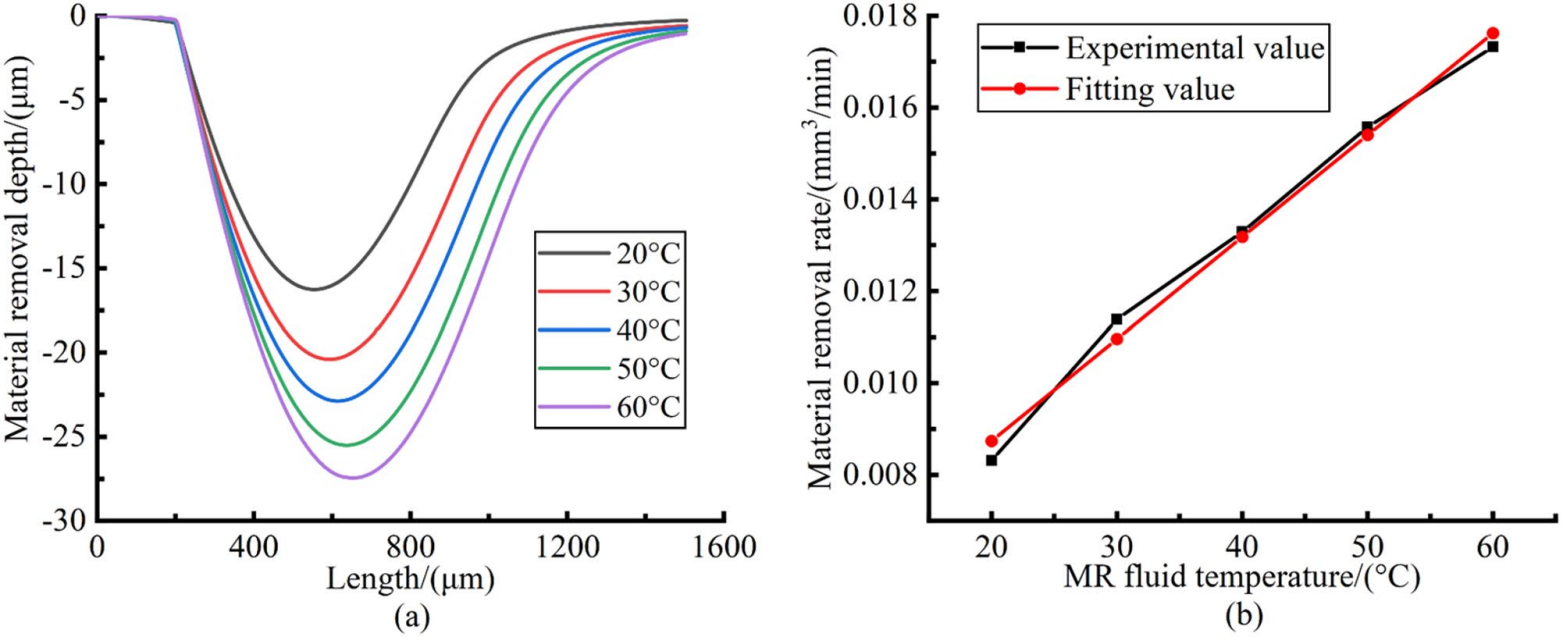
The results of verification experiment. (**a**) The section contour of the polished pit; (**b**) The change curve of material removal rate.

With the MR fluid temperature increases from 20 to 60 °C, the maximum removal depth of teardrop-shaped polished pits increases from 13 to 54 μm. Under different MR fluid temperatures, the shape of polished pits is similar with the distribution zone shape of material removal rate in Fig. 8. The position of the maximum material removal rate is located in the center of the polished pits, which is consistent with the theoretical calculation results in “Distribution zone of material removal rate”. This proves that after the MR fluid temperature increases, the convergence of surface shape accuracy in the material removal process can be maintained well. The section contour of the annular polished pit and the material removal rate of each annular polished pits are shown in Fig. 11.

It can be seen from the Fig. 11a that the position of the maximum material removal rate is still at the center of each polished pit. This suggests that the convergence of surface shape accuracy during polishing can still be maintained well. As shown in Fig. 11b, with the increase of MR fluid temperature, the material removal rate continues to increase. The material removal rate can be improved by 108.4% by increasing the MR fluid temperature from 20 to 60 °C. Experimental results suggest that the promoting effect of increasing polishing relative velocity on the material removal rate exceeds the inhibitory effect of decreasing shear stress, thus improving the material removal rate. Besides, by fitting the change curve of material removal rate, it is found that there is an approximate linear relationship between material removal rate and temperature, as shown in Eq. (12).

12 $$ MRR = 2.2206t/10000 + 0.0043 $$

where *MRR* is the material removal rate (mm^3^/min), *t* is the MR fluid temperature (°C).

**Figure 12:**
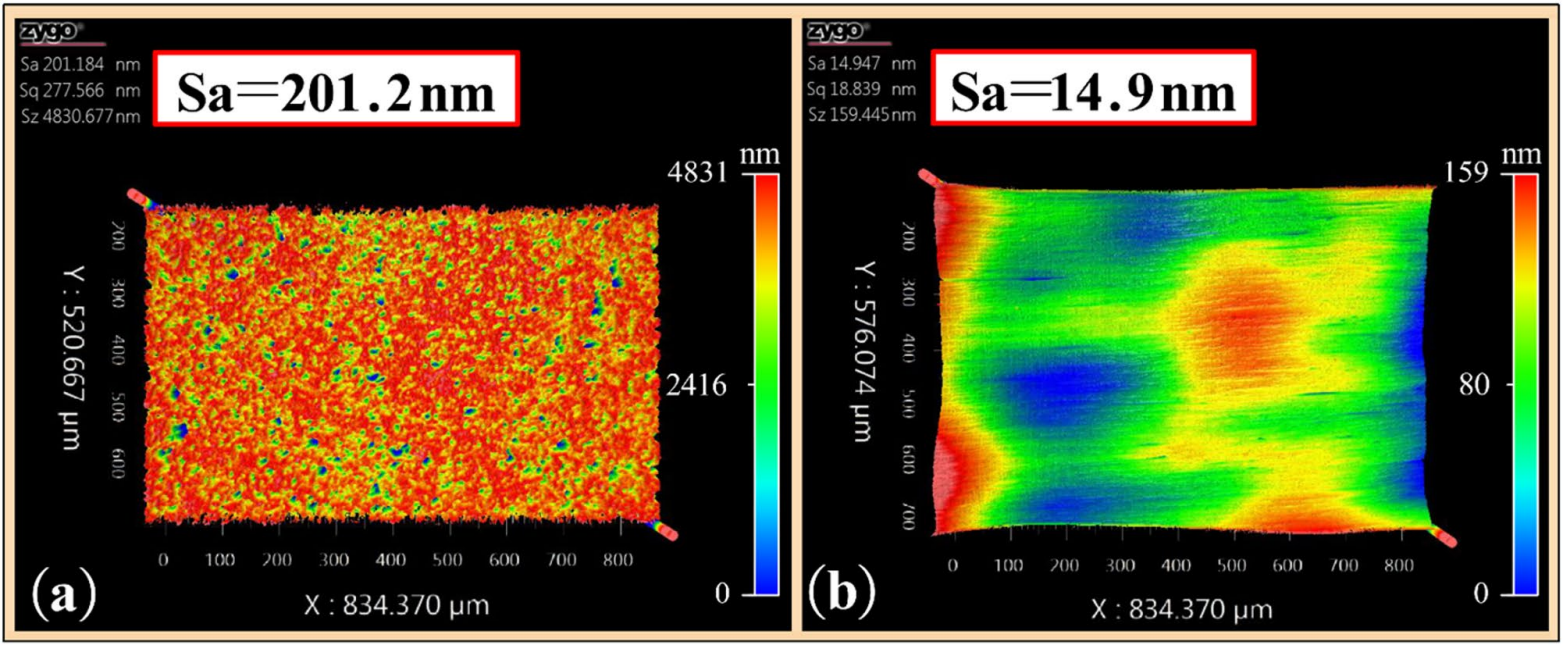
Surface roughness. (**a**) Roughness of initial surface; (**b**) Roughness of polished surface.

The coefficient of determination *R*^2^ of the fitting equation exceeds 0.99, showing that the equation is in good agreement with the change curve of material removal rate. Under the same processing conditions, the material removal rate at different MR fluid temperatures can be predicted by this equation. Keeping the process parameters unchanged, a section of smooth surface was polished on the rough surface at the MR fluid temperature of 60 °C, as shown in Fig. 9f. The measured surface roughness is shown in Fig. 12.

It can be seen from Fig. 12 that the surface roughness Sa reduces from 201.2 to 14.9 nm after polishing. This shows that under the condition when the MR fluid temperature is 60 °C, it can not only maintain the high material removal rate, but also obtain the smooth high-quality polished surface.

## Conclusion

Through theoretical analysis and experimental verification, it is found that increasing the MR fluid temperature can improve the efficiency of permanent magnet small ball-end MR polishing. The research results are summarized as follows.

1. In the range of 20–60 °C, the initial viscosity decreases with the increase of MR fluid temperature, which can improve the fluidity of MR fluid film. The magnetization intensity of CIP is very stable and hardly affects the material removal rate. The magnetic field intensity of the polishing head decreases with the increase of temperature, resulting in decreasing the yield stress of MR fluid.
2. Increasing the MR fluid temperature can increase the polishing relative velocity, but also reduce the shear stress. The increase of polishing relative velocity has a promoting effect on improving the material removal rate, but the decrease of shear stress has an inhibitory effect. Experiment results show that the promoting effect can exceed the inhibitory effect, so as to improve the material removal rate.
3. The material removal rate continues to increase with the increase of MR fluid temperature, and the convergence of surface shape accuracy in the material removal process can be maintained well at different temperatures. When the MR fluid temperature is 60 °C, the polishing removal efficiency is improved by 108.4%, and the polished surface roughness Sa can reach 14.9 nm.

In conclusion, the polishing removal efficiency can be improved by increasing the MR fluid temperature. This method can be applied to practical production to shorten processing time and increase production.

## Data Availability

The datasets generated during and/or analysed during the current study are available from the corresponding author on reasonable request.
